# Impact of Buffer, Protein Concentration and Sucrose Addition on the Aggregation and Particle Formation during Freezing and Thawing

**DOI:** 10.1007/s11095-018-2378-5

**Published:** 2018-03-19

**Authors:** Astrid Hauptmann, Katja Podgoršek, Drago Kuzman, Stanko Srčič, Georg Hoelzl, Thomas Loerting

**Affiliations:** 1 Institute of Physical Chemistry, University of Innsbruck, Innrain 52c, 6020 Innsbruck, Austria; 2 Lek Mengeš, 1234 Mengeš, Slovenia; 3 Faculty of Pharmacy, University of Ljubljana, 1000 Ljubljana, Slovenia; 4 Sandoz GmbH, 6336 Langkampfen, Austria

**Keywords:** buffer and mAb formulation, cryomicroscopy, freezing, particle formation, thawing

## Abstract

**Purpose:**

This study addresses the effect of freezing and thawing on a therapeutic monoclonal antibody (mAb) solution and the corresponding buffer formulation. Particle formation, crystallization behaviour, morphology changes and cryo-concentration effects were studied after varying the freezing and thawing rates, buffer formulation and protein concentration. The impact of undergoing multiple freeze/thaw (FT)-cycles at controlled and uncontrolled temperature rates on mAb solutions was investigated in terms of particle formation.

**Methods:**

Physicochemical characteristics were analysed by Differential Scanning Calorimetry whereas morphology changes are visualized by cryomicroscopy measurements. Micro Flow Imaging, Archimedes and Dynamic Light Scattering were used to investigate particle formation.

**Results:**

Data retrieved in the present study emphasizes the damage caused by multiple FT-cyles and the need for sucrose as a cryoprotectant preventing cold-crystallization specifically at high protein concentrations. Low protein concentrations cause an increase of micron particle formation. Low freezing rates lead to a decreased particle number with increased particle diameter.

**Conclusion:**

The overall goal of this research is to gain a better understanding of the freezing and thawing behaviour of mAb solutions with the ultimate aim to optimize this process step by reducing the unwanted particle formation, which also includes protein aggregates.

## Introduction

The development and optimization of freezing and thawing (FT) processes of therapeutic proteins as pharmaceutical products are among the challenges encountered by the pharmaceutical industry. Even though the process is used routinely in industry, it comes with some disadvantages. Compared with room temperature storage the FT-process is time-consuming and costly and may result in quality loss of the protein (1–3). The benefits outweighing these disadvantages include foam prevention and mechanical stress reduction during transportation, decrease of bioburden and microbial growth during storage and enhancement of stability of the product by slowing down any kind of degradation reaction rates that could lead to aggregation (4). Like all other therapeutic proteins, monoclonal antibodies (mAb) may undergo structural changes during FT processes which lead to protein aggregation, misfolding/unfolding, loss of biological activity or enhanced immune response in patients (5–7). The aggregation process is generally poorly understood (8) and may have several causes, such as cryoconcentration (9), contact with interfaces (1,10,11), cold denaturation (12,13), and many others (14–17).

In this work the focus lies on the systematic search for a good buffer formulation and freeze-thaw protocol for an in-house chimeric monoclonal antibody to reduce aggregation, which is a common goal of many pharmaceutical industries (18). To this end, the influence of protein concentration as well as addition of sucrose as stabilizer was scrutinized. Furthermore, the impact of freezing and thawing rates and the impact of several freeze/thaw cycles on particle number were studied using a range of methods detailed below.

It is, thus, one of the goals of this study to minimize formation of aggregates, which we synonymously also call particles. Here, »particles« is an umbrella term for all kinds of agglomerates regardless whether the particles are originating from the protein monomer or from other sources. Aggregates may be categorized according to size, type of intermolecular bonds, reversibility, morphology or hydrophobicity (19). When classified according to their size protein aggregates are subdivided into visible (>100 μm), micron (1–100 μm), submicron (100–1000 nm), and nanometer particles (<100 nm) (20). Besides aggregation and/or denaturation of the protein, FT-processes can also lead to an accumulation of leachables (21,22) and formation of sub-visible particles (1,23,24). Sub-visible protein particles (typically 0.1–10μm) are too big to be analyzed by size-exclusion chromatrography (SEC) but too small to be visible by an unaided eye (7). These particles could be the potentially most immunogenic class of protein aggregates and may act as nuclei (25) and cause formation of larger particles over time (5,26). In order to detect and assign aggregates/particles to the categories mentioned above we have employed Micro Flow Imaging (MFI) and Dynamic Light Scattering (DLS) (27–29). MFI is used for detecting particles in size ranges from 2 to 300 μm and DLS to detect and characterize soluble aggregates on a length scale of ca. 1–800 nm (19,28).

In order to avoid aggregation in the frozen solution the storage temperature should be chosen low enough, so that the solution as a whole has turned into a solid. This is the case below the glass transition temperature of the freeze-concentrated solution (T_g_’) (30,31). Since T_g_’ of a protein solution is determined by the concentration and nature of the cosolutes, buffer composition, pH and ionic strength it is necessary to establish an optimal formulation (32–36). Optical cryomicroscopy (OCM) and differential scanning calorimetry (DSC) are employed here to determine freezing temperatures, supercooling or T_g_’ from the latter and an analysis of the distribution of solid and liquid upon freezing and thawing from the former. In other words, the effects of cryoconcentration are studied using the combination of a thermal and optical image method. It is useful to distinguish between micro- and macro-cryoconcentration in this context (37).

**Fig. 1:**
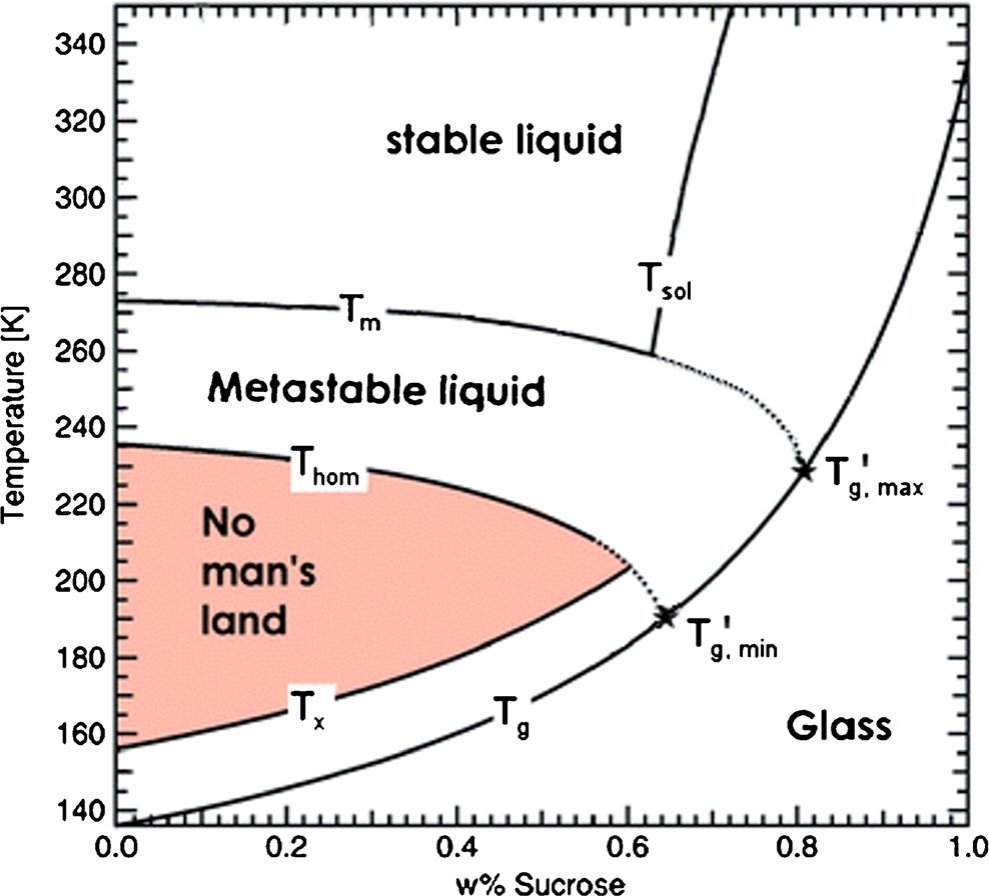
State diagram of sucrose-water, detailing melting temperature T_m_, homogeneous nucleation temperature T_hom_, glass transition temperature T_g_ and crystallization temperature T_x_. The freeze-concentrated solution will show a glass transition temperature anywhere between T_g,min_’ and T_g,max_’. Data taken from reference (38).

Microscopic scale cryoconcentration is based on the unavoidable dehydration of the liquid phase when water molecules form ice crystals. Solutes are trapped in the interdentritic space and increasingly freeze-concentrate until they reach the maximally freeze-concentrated solution (MFCS) (38). In a state diagram, such as the one for sucrose-water depicted in Fig. 1, the MFCS is given by the intersection of the melting (T_m_) and glass transition (T_g_) lines at the point denoted T_g,max_’. This is the case at roughly 80 wt% in Fig. 1 and implies that freezing takes place at the melting line. In real experiments, however, supercooling plays an important role, and the solutions might remain in the liquid state down to the homogeneous nucleation temperature (T_hom_ in Fig. 1). In this case, the dehydration incurred upon ice freezing may result in a freeze-concentrated solution of lower T_g_, which is less concentrated than the MFCS. In the example of sucrose in Fig. 1 this may result in vitrified solutions of 64 wt% sucrose, as defined by the intersection of T_hom_ and T_g_ at T_g,min_’.

Macroscopic scale cryoconcentration on the other hand happens when solutes or protein are progressively pushed into a certain direction in the bulk due to the growing ice crystal front of the water and the exclusion from the solid-liquid interface (22). Macroscopic scale cryoconcentration is very hard to control since it depends on a large number of parameters, including the size of the sample, the temperature gradients in the sample, etc. When both micro- and macro-cryoconcentration occur two distinct freeze-concentrated solutions, FCS1 and FCS2, may be observed (39).

The cooling rate has a large influence on the degree of supercooling, and consequently the concentration of FCS1 and FCS2 as well as the number of ice nuclei. These factors together in turn affect the formation of aggregates. The slower the freezing the more freeze-concentration on the macroscopic scale plays a role (22), which may lead to partial unfolding and loss of protein activity (16,40). Fast freezing may lead to formation of a very large number of small ice dendrites, therebye increasing the size of the ice-liquid interface (16,40) and creating a large number of interdendritic spaces (31). Also this may be unfavorable for the protein (17,41), but was also suggested to favor protein stability due to more uniform protein distribution (31,42).

Slow thawing may result in cold-crystallization, i.e., growth of small ice nuclei to small ice crystals from devitrified freeze-concentrated solution above T_g_’ (22). Consequently, the optimal freezing-thawing rate cannot be stated in general, but may differ from solution to solution. Usually slow freezing and faster thawing rates are preferred (16,17,22). In order to minimize the damaging effects cryoprotectants/cryostabilizers maybe helpful (30,43). This strategy is often used for protein solutions (3,16,24,44). Usually formulation additives such as sugars, aminoacids, polyvalent alcohols, oligosaccharides or polymers are used in order to stabilize the protein conformation and to prevent its adsorption on the surface of ice crystals. It is, thus, of importance to optimize the formulation, too. Sucrose and trehalose are popular examples for such additives in protein solutions. In this context, it is important to note that these additives should not crystallize themselves but turn into the glassy state. Furthermore, it is of importance to control the solution pH – at low pH sucrose in solution can cause glycation (non-enzymatic glycosylation) of the monoclonal antibody (45,46). This is usually done by employing buffers, such as phosphate or citrate, which also need to stay amorphous upon freezing and thawing (47). For this reason we here study the freezing of a buffer solution containing a range of protein concentrations with or without the addition of sucrose as cryoprotectant.

## Material and Methods

In this study we are focusing on the aggregate formation of a monoclonal antibody (mAb) formulation. Protein solutions in the concentration range 1–30 mg/ml were embedded in a 25mM sodium citrate buffer (pH 6.5) with or without addition of 125mM sucrose. The following studies were done:

### Freezing Box and Celsius-S3 Studies

**Fig. 2:**
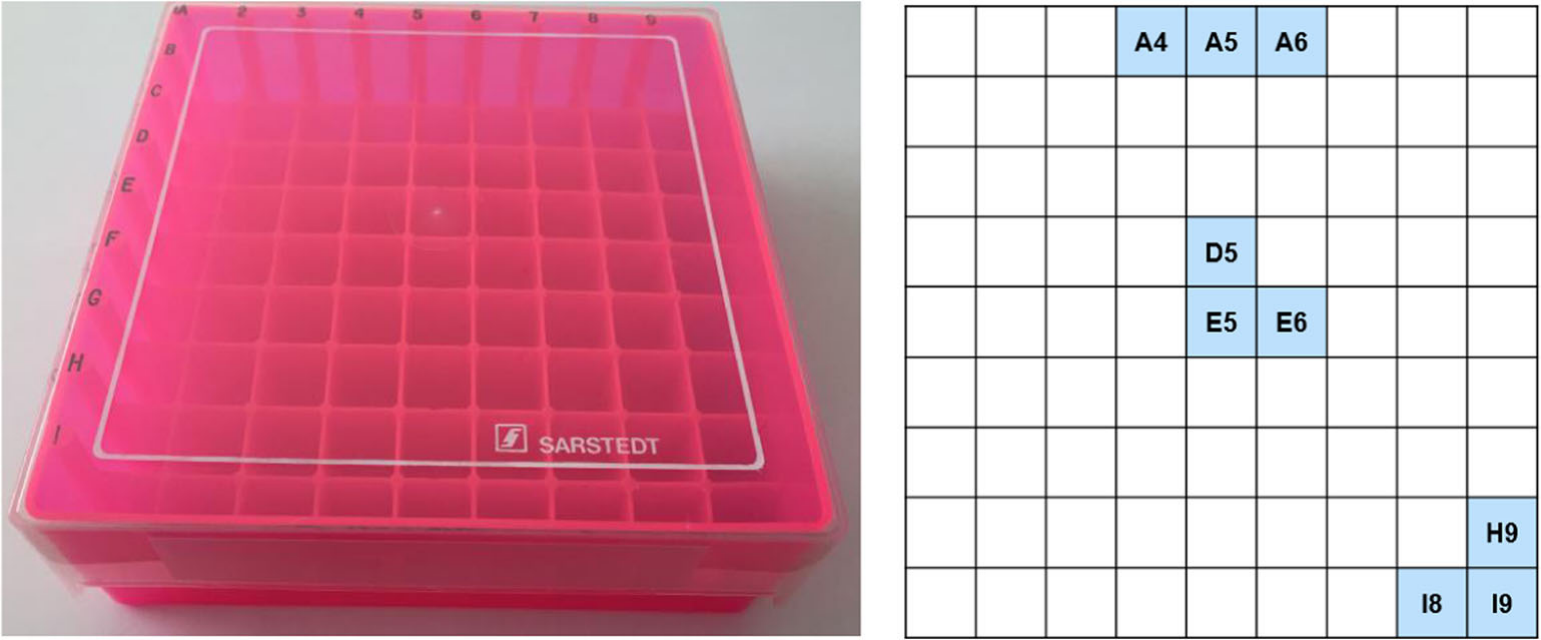
Freezing box and the positions of the samples.

In order to evaluate the role of freezing/thawing rate and the number of freeze cycles on aggregate formation samples with a protein content of 1 mg/ml were placed in a freezing box at different positions between vials filled with water (Fig. 2). The freezing box was placed in a -80°C freezer. Temperatures recorded during freezing have shown the typical shape, including a linear cooling ramp in the liquid state, a freezing plateau and another linear cooling ramp in the solid state. For samples placed in the middle of the box the liquids were cooled at 1.1 K/min, and the freezing plateau of constant temperature lasted for 9.2 min. For samples placed at the corner, the cooling rate was found to be higher (3.9 K/min), and the freezing plateau only lasted for 2.4 min. A supercooling of −4.7°C was observed. After having cooled to below T_g,min_’ the samples were thawed by bringing them to room temperature again. This procedure was repeated up to five times.

In another set of experiments the concentration of mAb in the formulation was varied from 1 mg/ml to 30 mg/ml, with or without the addition of sucrose. Samples were frozen and thawed in the Celsius®–S3 Benchtop System (Sartorius, Germany) (simulating the freezing protocol of our production scale). The system is temperature controlled by a cooling liquid (Syltherm HF®HTF) which flows circularly around the cooling chamber. Temperatures were controlled using the computer program Cryopilot and 10 thermocouples (Pt 100). The sample holder, originally made to freeze and thaw plastic Celsius-Paks, was custom made for placing 1 ml, 1.5 ml and 2 ml Nunc CryoTubes or 4–5 ml PETG vials. For particle characterization the sample was homogenized by turning the vial 10 times carefully by hand followed by letting the sample settle for 1 h at ambient temperature. After turning the sample vials for 10 times again the samples were aliquoted and measured by using MFI and DLS.

### Differential Scanning Calorimetry (DSC)

DSC measurements were conducted on a DSC 8000 (Perkin-Elmer, USA). Hereby, around 30 μl of sample was loaded into an Al crucible, hermetically sealed and transferred to the device at ambient temperature. An empty aluminium pan was used as a reference. Starting from 10°C the sample was cooled to −90°C, equilibrated for 5–10 min and re-heated at rates of 1°C/min, 2°C/min, 5°C/min, 10°C/min, 20°C/min and 40°C/min. For the calibration at subzero temperatures the recommended transitions (48) in cyclopentane, cyclohexane, indium, and adamantane were used in order to ensure correct tansformation temperatures with an accuracy of ±1°C. An accuracy of ±0.1 J/mol K in latent heat and change in heat capacity can be achieved due to an calibration using sapphire.

### Optical Cryomicroscopy (OCM):

Images of frozen buffer solution with or without sucrose were taken by an optical microscope BX-51 (Olympus Corporation, Japan) in combination with a temperature controlled cryostage LTS420 (Linkam Scientific Instruments, UK). With the Linkam-cryostage subzero temperatures down to −196°C can be reached by using liquid nitrogen as cooling medium. A drop of sample (approx. 0.5 μl) was placed on the object plate and either directly positioned into the cryo-chamber without covering, or firstly enclosed by a cover glass slip before analyzing in order to prevent evaporation. Loaded at ambient temperatures, samples were next frozen to −80°C at rates of 1°C/min, 2°C/min, 5°C/min, 10°C/min, 20°C/min, 30°C/min and 40°C/min and analyzed isothermically by using standard transmitted light with a crossed-polarized filter. Crossed-polarized light can be used for visualization of structural changes, crystal sizes and ortientation in the droplet. Morphology changes in the frozen form were determined by calibrating an ULWD 50× objective (Olympus Corporation, Japan) and using the Linksys 32 software. The sample droplet had to be analyzed as a thin layer, which could be achieved by using a top glass positioned on the sample droplet while measuring. The downside of that method had been the accumulation of ice crystals formed onto the top glass especially at lower rates which originated from the humid environment of the cryo chamber and are a product of condensation.

### Micro-Flow Imaging (MFI)

MFI System MFI 5100 (ProteinSimple, Canada) was used to capture particle images as the sample passes continously through a 400 μm flow cell in front of a CCD camera. Micron sized and visible particles and protein aggregates (2–300 μm) can be depicted in thousands of frames per minute and extracted by the MFI View System Software where they are categorized. The Software provides specific information about the properties like size, number (counts/ml) and morphology of the particles (49). 5% PCC (Thermo Fischer Scientific, USA) solution, a mixture of anionic and nonionic surfactants, and milli-Q-water was flushed through the MFI device before and after each measurement in order to keep the system clean and retain a clean base line. 1.5 ml of undiluted protein solution were injected into the flow cell (0.2 ml for pre-run/purge volume and 0.615 ml for analysis) and analysed using a flow rate of 0.22 ml/min.

### Archimedes

Archimedes (Malvern, United Kingdom) was equipped with Hi-Q microsensor (Malvern) and was used for particle characterization in the size range from 200 nm to 5 μm. The system was controlled by the Archimedes Version 1.20 Software. Before every analysis blanks (UPW/ milli-Q-water) were measured to ensure a clean system. This is defined by the blank showing less than 20 particles after 10 min of analysis. Before sample measurement the system automatically determines the limit of detection (LOD) which can be subsequently changed for results comparison and processing. Each sample was loaded for 30 s and was then analysed (100 nl of sample solution). Between each sample measurement the system was flushed with milli-Q-water.

### Dynamic Light Scattering (DLS)

A Zetasizer APS (Malvern, United Kingdom) supported by the Zetasizer Software DTS software was used to measure the size and distribution of nanometer and submicron particles. The device automatically measures liquid samples in microtiter plates. After thawing each sample (100 μl) was analysed five times at ambient temperatures and afterwards discarded. In addition to protein samples the standard of latex spheres size 60 nm and 200 nm (Duke standards™, Thermo Scientific) was also measured before and after analysis of samples to ensure correct operation. The plate filled with samples was protected with aluminium foil during the analysis.

## Results

### Particle Numbers and Sizes

**Fig. 3:**
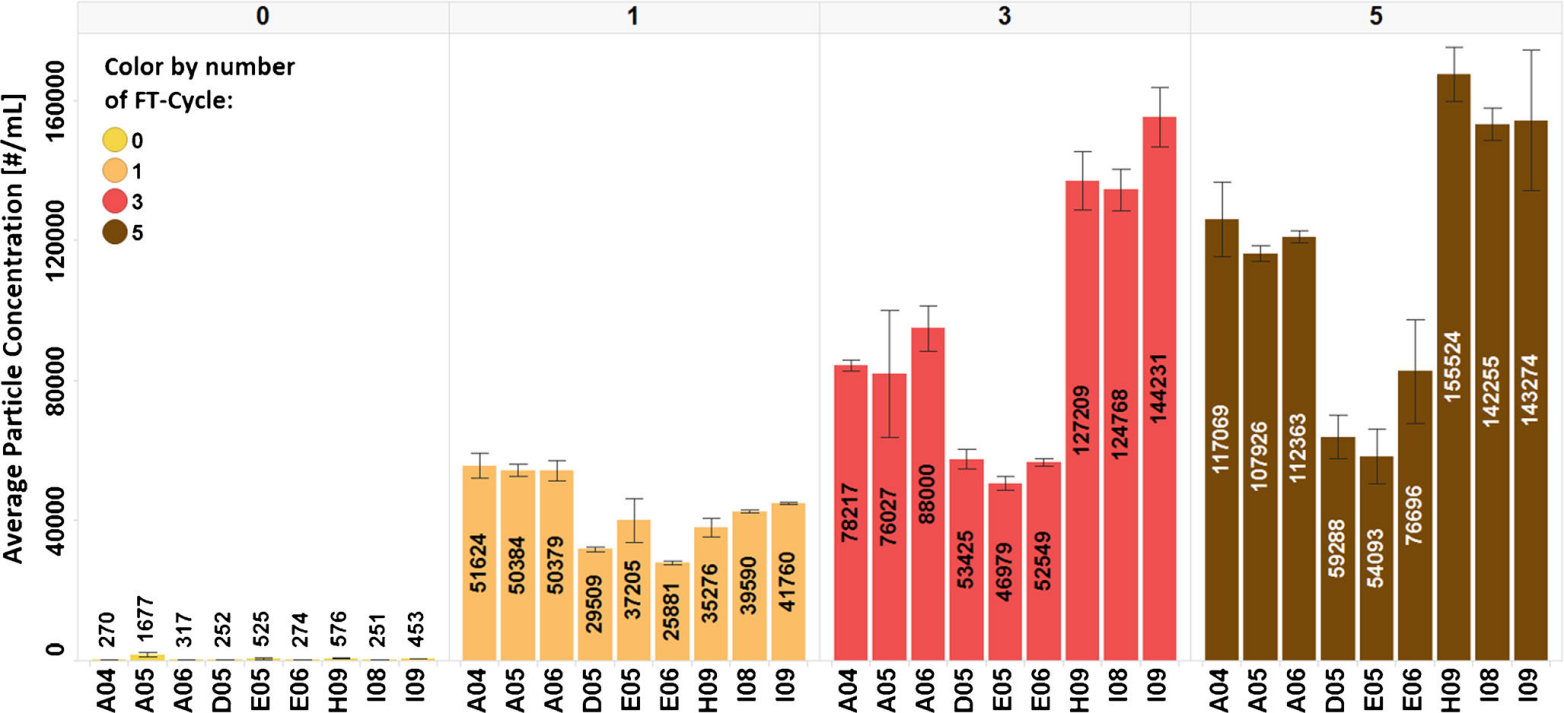
MFI data for average particle number in the mAb formulation (mAb + 25 mM sodium citrate buffer) after 0, 1, 3 and 5 F/T cycles according to the vial position in the freezing box (see Fig. 2).

**Fig. 4:**
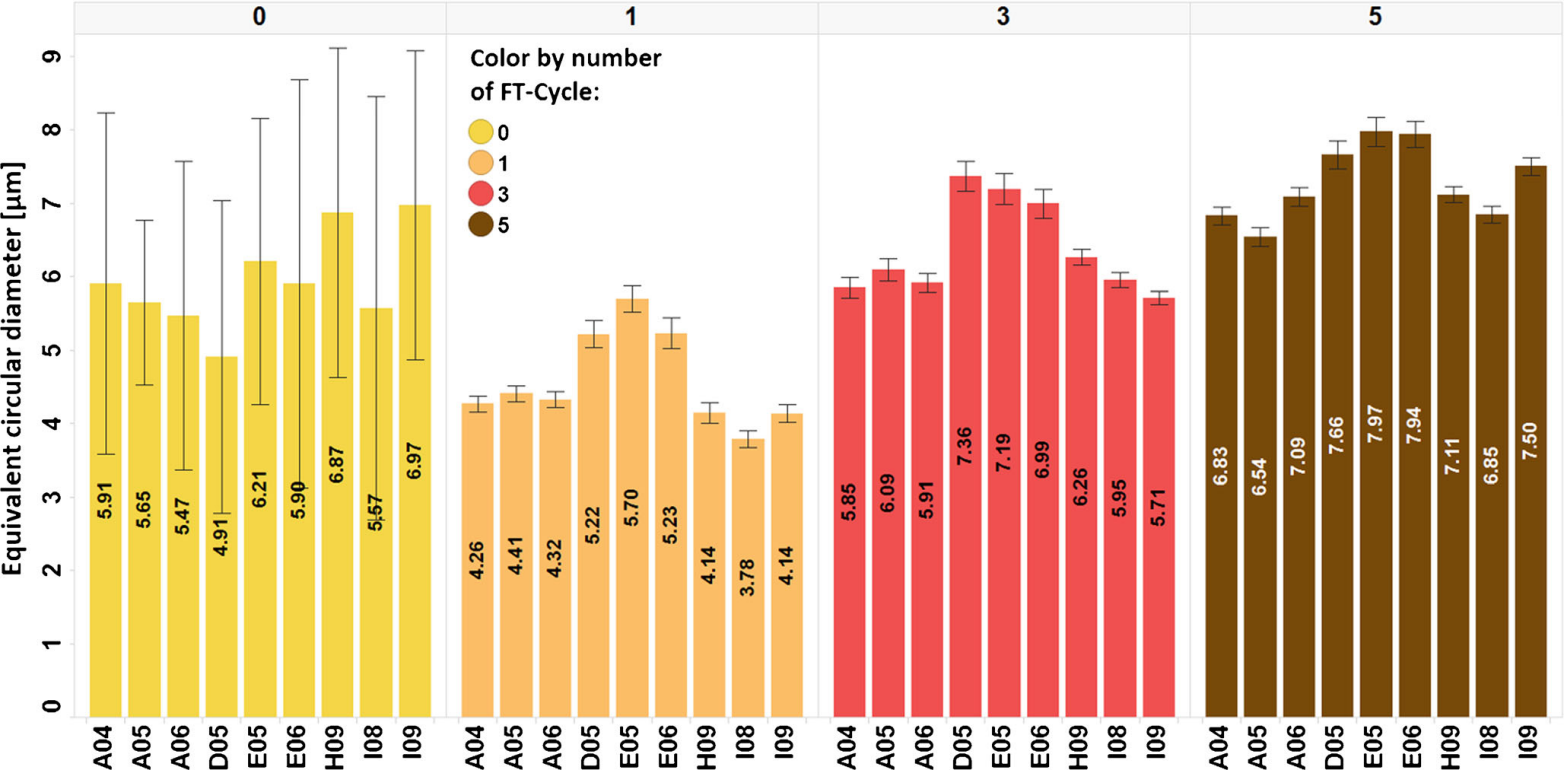
MFI data of the average particle size in the mAb formulation (mAb + 25 mM sodium citrate buffer) after 0, 1, 3 and 5 F/T cycles according to the vial position in the freezing box (see Fig. 2). Since the number of particles is small for cycle 0 (Fig. 3) one or a few larger particles have a big influence on the circular diameter and error bars of equivalent circular diameter (SE = SD/√N).

First, we show the influence of the number of F/T cycles on the number (Fig. 3) and circular equivalent diameter (Fig. 4) of particles forming in 1 mg/ml protein solution without sucrose in 25 mM sodium citrate buffer. MFI data (Fig. 3) show the progression of particle numbers with F/T cycles. Each additional F/T-cycle leads to an increase of particle number and hence to aggregate formation in the sample. After the fifth cycle the number of particles is half in the samples positioned in the middle of the box *vs*. the edges (Fig. 3), but about 15% bigger in terms of diameter (see Fig. 4). That is, especially samples placed at the edges of the freezing box are affected mostly by the repeated F/T cycles. This clearly demonstrates that temperature gradients play an important role for the formation of particles. Smaller temperature gradients found in the middle of the box are favorable to avoid high particle numbers, but will lead to larger particles.

**Fig. 5:**
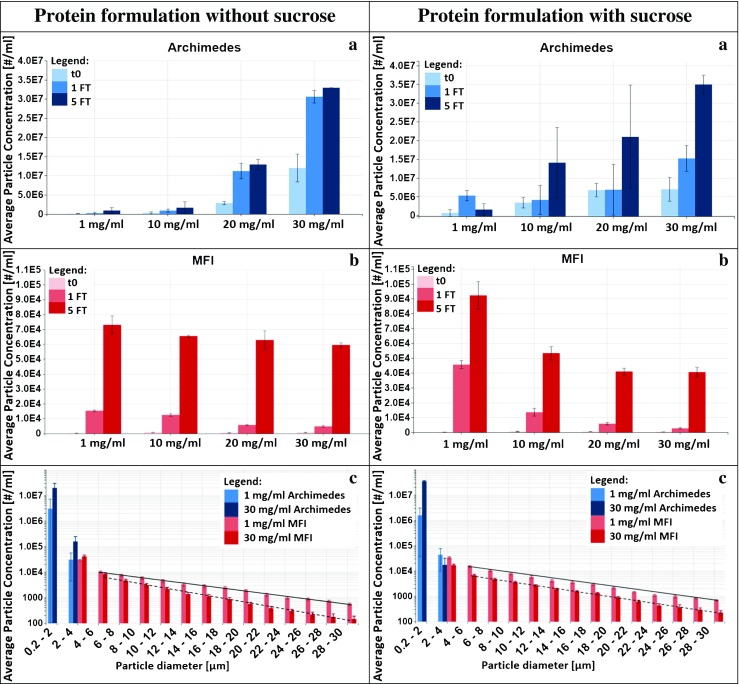
Data retrieved from Archimedes (**a**) MFI (**b**) and the corresponding size range distribution (**c**) of samples of 1 mg/ml, 10 mg/ml, 20 mg/ml and 30 mg/ml mAb in 25 mM sodium citrate buffer with (right column) and without 125 mM sucrose (left column).

Samples with different protein concentration were analyzed by Archimedes and MFI (see Fig. 5) and DLS (see Table I) with and without sucrose being added. Higher protein concentration results in an increased number of small particles detected by Archimedes (ranging from 200 nm to 5 μm, Fig. 5a). However, simultaneously it results in a decreased number of larger particles (micron and visible) in the size range from 2 μm to 300 μm detected by MFI (Fig. 5b). Figure 5c compares the influence of the initial protein concentration directly: higher concentration increases the number of small particles up to 5 μm, but decreases the number of larger particles.

**Table I:** Polydispersity Index (PdI) and Hydrodynamic Diameter 2r Retrieved from DLS Measurements on Samples of 1 mg/ml, 10 mg/ml, 20 mg/ml and 30 mg/ml Protein in Sodium Citrate Buffer with and without Sucrose

		1 mg/ml		10 mg/ml		20 mg/ml		30 mg/ml	
		-sucrose	+sucrose	-sucrose	+sucrose	-sucrose	+sucrose	-sucrose	+sucrose
t_o_	PdI	0.005	0.193	0.016	0.038	0.047	0.038	0.063	0.062
	2r [nm]	11.4	11.2	12.8	13.8	14.5	15.5	16.2	17.1
1FT	PdI	0.006	0.206	0.026	0.041	0.049	0.053	0.065	0.062
	2r [nm]	11.3	11.2	12.7	13.6	14.5	15.4	16.3	17.1
5FT	PdI	0.021	0.172	0.042	0.047	0.063	0.078	0.079	0.086
	2r [nm]	11.4	11.4	12.8	13.7	14.8	15.5	16.7	17.2

The influence of the number of FT-cycles is best seen in Fig. 5a, b, in which the three bars detail the number of particles before the first, after the first and after the fifth FT-cycle. Archimedes measurements reveal that the particle numbers increase after the first FT-cycle significantly, but remain almost constant between the first and fifth FT-cycle, regardless of the protein concentration. In contrast, MFI results indicate a drastic increase of micron/visible particles from the first to the fifth FT-cycle. That is, the number of larger particles increases with the number of FT-cycles, but the number of the small particles remains invariant. Focusing on particle sizes bigger than 4 μm, the decrease of particle number in each size range shows a linear trend. The trend line of samples with 30 mg/ml protein concentration has a steeper slope than the one from samples with 1 mg/ml (see Fig. 5c). Since the number of particles is plotted logarithmically, the linear behaviour implies particle numbers decreasing exponentially with size.

The influence of the addition of 125 mM sucrose to the solution can be seen by comparing the left and right panels in Fig. 5a–c. On first look sucrose does not seem to have a very large influence on the number of particles. On closer inspection it can be noticed that the number of particles is actually increased for the 1 mg/ml protein solutions, but decreased for the 30 mg/ml solutions. This is true especially for the small particles after the first freezing cycle (Fig. 5a). After the fifth cycle, however, the beneficial effect of sucrose on the small particles has disappeared again. For the larger particles addition of sucrose, results in a reduction of about one third (Fig. 5b, 30 mg/ml) even after the fifth cycle.

The polydispersity index (PdI) and the hydrodynamic diameter 2r as obtained from DLS measurements are listed in Table I. The PdI increases dramatically for samples with 1 mg/ml protein after the addition of sucrose, but is barely affected for all other solutions by sucrose. Thus, higher protein concentration enforces monodispersity. Except for the 1 mg/ml protein solution the PdI increases slightly after 5 FT-cycles. At the same time addition of sucrose increases 2r by about 1 nm, compared to the solutions without sucrose. Again the exception is the 1 mg/ml protein solution, for which only the PdI is increased massively, whereas 2r remains unaffected by the addition of sucrose. However, 2r increases systematically with protein concentration, from 11.2 nm at 1 mg/ml to 17.2 nm at 30 mg/ml (Table I). The hydrodynamic diameter of the mAb monomer is known to be 11.2 nm. This value was measured in a protein concentration of 1 mg/ml at ambient temperatures and also in accordance with the value predicted by using the Hydropro software (50) on the crystal structure of an intact IgG mAb (51).

### Cryoconcentration Effects

**Fig. 6:**
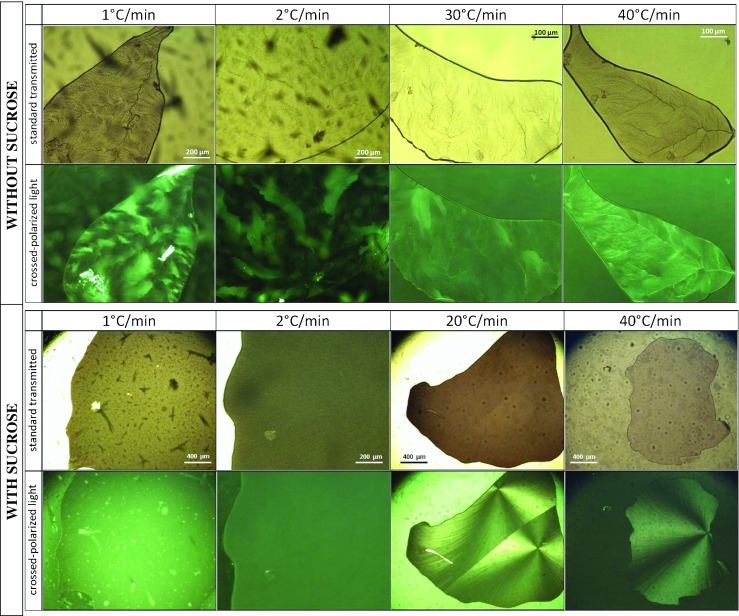
OCM images of 25 mM sodium citrate buffer without (top two rows) and with (bottom two rows) sucrose at −80°C after cooling with the indicated rates. Droplet was placed on an object slide and covered by a glass while measured.

The influence of cooling rates on the morphology of the frozen samples was studied on individual droplets of millimeter size using OCM experiments. OCM images allow for resolution of freezing-induced morpholgocial changes on the μm-length scale. Figure 6 shows the changes induced upon cooling buffer solutions without (top two rows) and with sucrose (bottom two rows), respectively. Generally, pure buffer without sucrose shows many features after cooling to −80°C. Channels reminiscent of leaf-veins have grown throughout the droplet (Fig. 6, top row) and shade the transparent patches of ice crystals with darker nuances. These veins are a result of macro-cryoconcentration and contain freeze-concentrated buffer solution. When crossed-polarized light is applied (Fig. 6, second row) the orientation of the ice crystals and their edges, junctions and triple junctions become apparent through different tones of green. After slow cooling at 1 or 2°C/min relatively large ice crystal platelets of dimensions 100–300 μm can be defined and located precisely. The maximally freeze-concentrated solution (MFCS) is located mainly at the edges of the few larger crystals. After faster cooling at 30–40°C/min fading and/or loss of the platelet structures together with the formation of an extended network of veins that pass through the droplet is observed (Fig. 6, top rows). That is, the ice crystals are much smaller, and the MFCS is more widely and less heterogeneously dispersed for faster cooling rates.

Homogeneity increases even more in sodium-citrate buffer with 125 mM sucrose, which results in opacity of the droplet in the frozen state (Fig. 6, bottom two rows). Images taken after freezing at 1 or 2°C/min show a rather homogenously colored green surface through crossed polarizers. After freezing at 20 or 40°C/min star-shape like patterns appear when using crossed-polarized light. Two such stars are observed after cooling at 20°C/min, and only a single one after cooling at 40°C/min. We interpret the centers of these stars as individual nuclei, from which the ice crystal growth has started. Veins such as the ones in Fig. 6 (top row) are absent when sucrose is added. This implies that the FCS is much more homogeneously distributed. Note that the dark spots that appear both inside and outside the droplets are small ice crystals condensed from air on top of the cover glass.

**Fig. 7:**
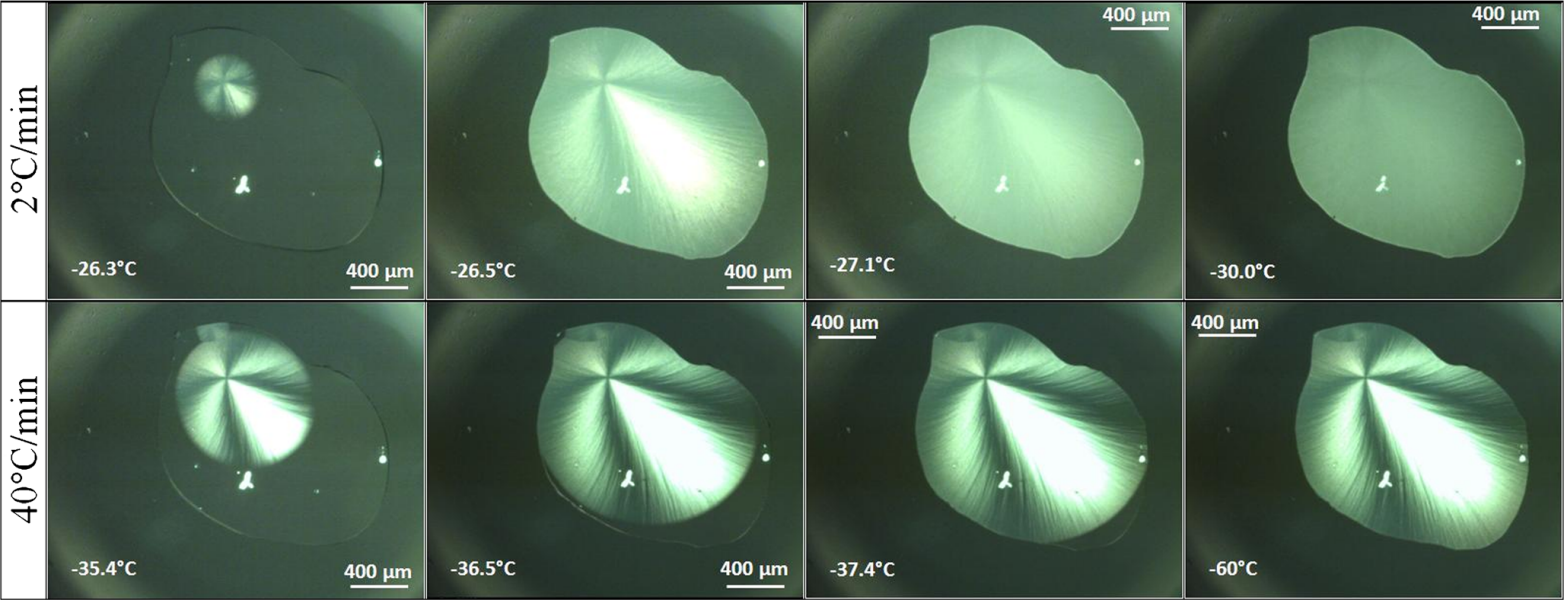
Snapshots of the first freezing process in solutions containing in addition 125 mM sucrose (left). Second freezing takes place for 2°C/min (top right), but vitrification for 40°C/min (bottom right).

The evolution of these star-shaped features with temperature in sucrose-citrate solution is detailed in Fig. 7. Visual inspection using the cryomicroscope shows that indeed the freezing process starts from a single spot, from a single ice nucleus, and it is followed by crystal growth directed towards the outer surface. The onset of the growth is observed at −26.3°C for 2°C/min cooling rate and at −35.4°C for 40°C/min (Fig. 7, left). The main difference of the freezing process at 2°C/min (Fig. 7, top row) and the one at 40°C/min (Fig. 7, bottom row) is that with faster rates the star-shape like crystal formation remains down to the lowest temperatures −60°C (Fig. 7, bottom right). When slower rates are applied the edges fade away (see Fig. 7, top right image) and the droplets appear as homogenously frozen. This homogenization is related to the second freezing event pertaining to the FCS dispersed homogeneously in between the rays of the star shape. This second freezing is only observed for slow cooling rates (see DSC data below).

**Fig. 8:**
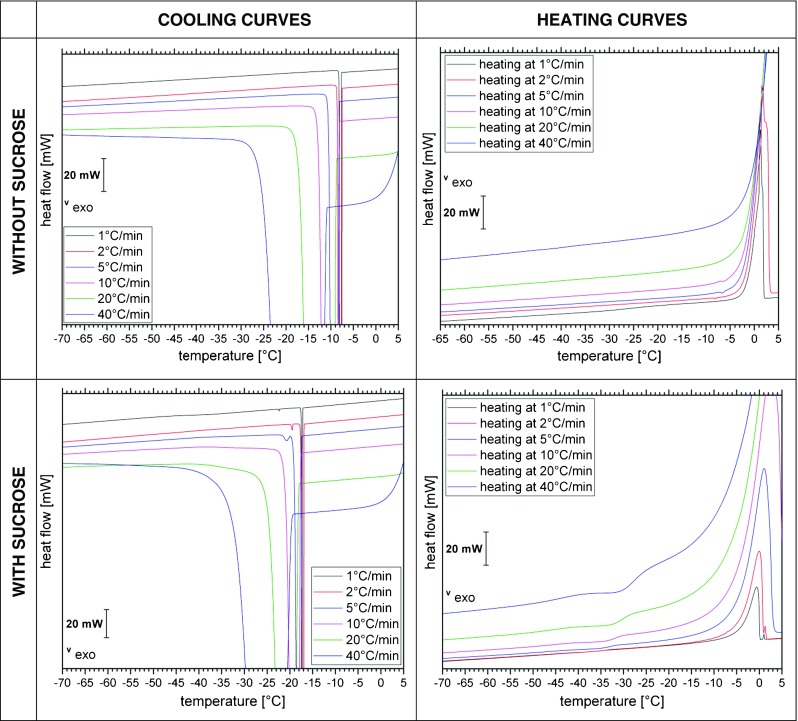
DSC cooling curves and heating curves of liquid aqueous solutions containing sodium citrate buffer without (top row) and with (bottom row) sucrose at different cooling and freezing rates recorded with the DSC 8000.

DSC measurements provide complementary thermal information – just like for the OCM study we have employed millimeter-sized droplets and cooling rates between 1 and 40°C/min. From the calorigrams shown in Fig. 8 we extract freezing temperatures (T_f_) as well as glass transition temperatures of freeze-concentrated solutions (T_g_’) and list them in Table II. Figure 8 also shows the thermal effects incurred upon thawing the frozen sample (right).

**Table II:** Freezing Temperatures T_f_, Glass Transition Temperatures T_g_’ and Width of Freezing Peak of 25 mM Sodium Citrate Buffer with and Without Sucrose Determined by DSC Measurements

Cooling and Heating Rates	Buffer without sucrose			Buffer with sucrose		
	T_f_ [°C]	T_g_’ [°C]	width	T_f_ [°C]	T_g_’ [°C]	width
1 °C/min	[−7; −17]^*^	–	0.6 ± 0.1	[−10; −16.6]	−36	0.5 ± 0.1
2 °C/min	[−7; −16]^*^	–	1.0 ± 0.1	[−10; −16.3]	−36	1.0 ± 0.2
5 °C/min	[−7.5; −17]^*^	–	2.4 ± 0.2	[−10.3; −16.7]	−34	2.3 ± 0.5
10 °C/min	[−7.5; −16.2]^*^	–	4.3 ± 0.5	[−11.6; −17]	−32.5	4.3 ± 1.0
20 °C/min	[−8; −18]^*^	−40	7.9 ± 0.9	[−12.2; −17.1]	−32	7.5 ± 1.9
40°C/min	[−9.7; −19]^*^	−40	13.2 ± 1.0	[−13; −18.3]	−32	13.5 ± 2.0

T_f_ was obtained from DSC cooling scans, T_g_’ from the subsequent heating scan. Width is determined as difference of onset and end point of the freezing transition. T_f_ is defined by the onset of the exotherm. For a thermodynamically controlled transition the onset temperature is rate invariant, except for thermal lag of the instrument at fast rates. By contrast, the offset typically shifts with a change of rate since the width of the transition is governed by kinetics, i.e., the time it takes for the whole sample to undergo the phase transition

*T_f_ indicates the range of freezing temperatures obtained from repeated measurements

All DSC scans recorded upon cooling show at least one massive exotherm, indicating the freezing of ice from the solution. In some cases the freeze-concentrated solution (FCS) experiences a second freezing event, whereas in other cases the first freezing event is followed by vitrification at Tg’ (Fig. 8). The second freezing exotherm appears only for cooling rates of 1–5°C/min in the presence of sucrose (Fig. 8, top left). However, the second freezing exotherm is never observed without the addition of sucrose (see Fig. 8, bottom left). The second freezing event for slow cooling rates in the presence of sucrose is responsible for the homogenization below −27°C observed in Fig. 7 at 2°C/min. In the DSC experiment the onset for the second freezing event is found to be between −20°C and −25°C, in agreement with the observations in the cryomicroscope.

**Fig. 9:**
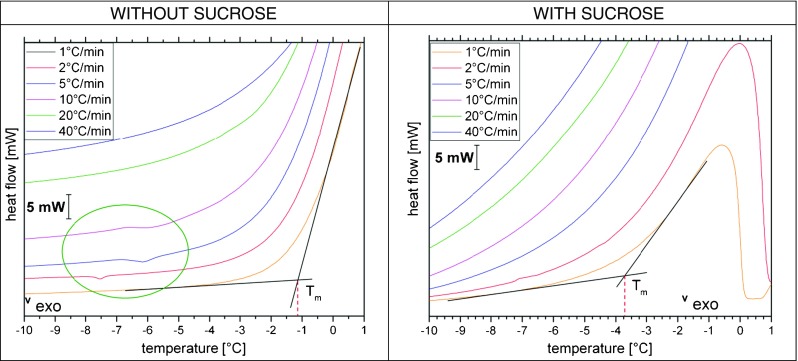
DSC heating curve at 1, 2, 5, 10, 20 and 40°C/min of liquid aqueous solutions containing sodium citrate buffer without (left) and with sucrose (right). The cooling rate to freeze the droplet is identical to the heating rate in the subsequent DSC heating scan.

For the first freezing event, i.e., crystallization of ice from the solution, T_f_ onset is seen between −7°C to −19°C for the pure buffer and between −10°C to −18.3°C for the buffer with sucrose (see Table II). Both freezing ranges are clearly below the thermodynamic freezing/melting temperature, which is revealed from the magnified DSC heating scan in Fig. 9 (right). From the tangent method on the slowest heating scan the equilibrium melting temperature T_m_ is evaluated to be −1.1°C without sucrose and −3.8°C with sucrose (see tangent intersection in Fig. 9). The difference between the two represents the melting point depression incurred because of the addition of sucrose. The supercooling (i.e., difference between T_f_ and T_m_) is found to be about 6–18°C without sucrose and 6–14°C with sucrose, but does not depend much on the cooling rates used here.

T_g_’ was determined using the “tangent” evaluation method (52) on magnified versions of the heating curves of the thermogram (not shown). The “tangent” evaluation has a precision of T_g_’ ± 3°C. The DSC heat flow is proportional to the heating rate employed. Since the glass transition pertains to the FCS in the veins, i.e., only a very minor fraction of the whole frozen sample, sensitivity is a key issue. Especially at small heating rates the sensitivity of our instrument is not high enough to resolve the glass transition for the freeze-concentrated buffer itself. Only for the heating scans at 20 and 40°C/min the heat flow signal clearly allows to resolve the increase in heat capacity associated with the glass transition at −40°C. The glass transition is easier to detect after sucrose addition, and so we are able to resolve it even on the slowest heating scans at 1°C/min. As expected for a transition that is kinetic in origin, it shifts to higher temperatures with increasing rates. We observe a shift of about +2°C when going from 1°C/min to 5°C/min (Table II). The addition of 125 mM sucrose shifts T_g_’ from −40°C to −32°C at the rate of 20°C/min, which is the standard rate typically used for the determination of glass transitions (52). In the sucrose-water state diagram (see Fig. 1) the MFCS is located at about 80 wt% sucrose, which shows a T_g_’ of about −46°C. Our T_g_’ is higher by about 10°C, which indicates that the MFCS state is not reached. From the sucrose-water state diagram we estimate that the vitrified FCS in the veins contains about 70 wt% sucrose.

In addition to the devitrification transition at T_g_’ we also observe a weak transition in the heating scans between −8° and −6°C (Fig. 9). We interpret these small effects as exotherms and as cold-crystallization of the devitrified FCS that takes place in the broad low-temperature tail of the melting peak. In other words, freezing and melting events take place simultaneously at this stage. The same effects were observed for solutions containing the protein in addition. DSC measurements of the mAb formulation seem to be very similar to the DSC curves of the buffer without sucrose (data not shown). The only difference noticeable is the broad T_g_’ and the absence of the kink in the heating curves when measuring mAb solution in buffer. Whereas, when sucrose is added to the buffer it leads to a shift of freezing temperatures and broadening of the melting peaks (Fig. 8).

**Fig. 10:**
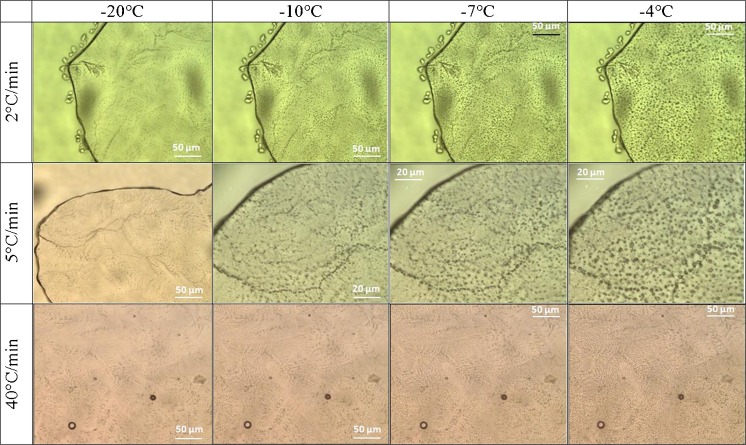
OCM images at the indicated temperatures recorded upon heating at the indicated rates for frozen 25 mM sodium citrate buffer solutions without sucrose.

**Fig. 11:**
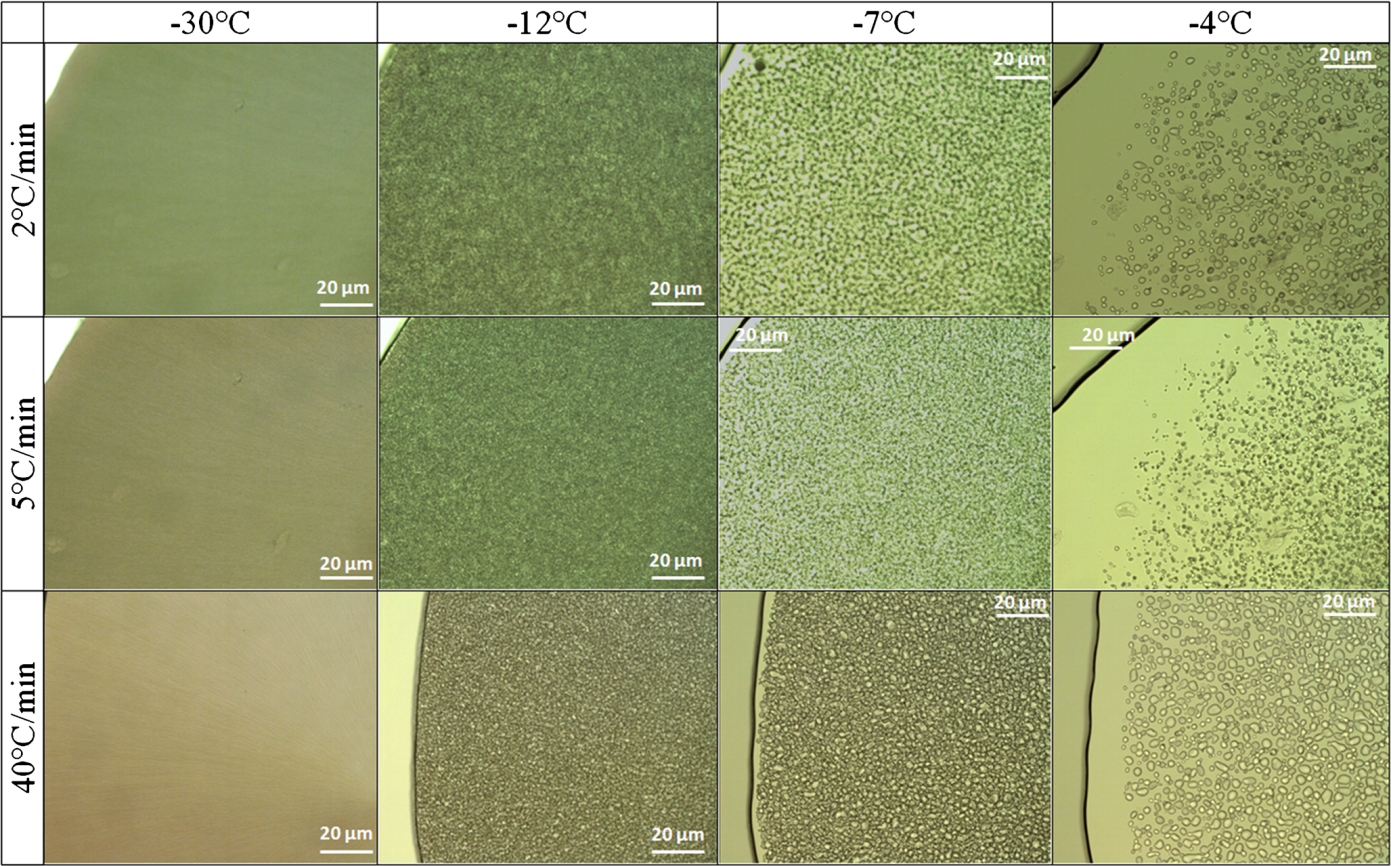
OCM images at the indicated temperatures recorded upon heating at the indicated rates for frozen 25 mM sodium citrate buffer solutions with sucrose.

The heating scans depicted in Fig. 9 are complemented with cryomicroscopy heating measurements shown in Fig. 10 (25 mM buffer without sucrose) and in Fig. 11 (25 mM buffer with 125 mM sucrose). The images were recorded upon heating at 2°C/min, 5°C/min and 40°C/min (right after cooling the solution droplets to −80°C at the same rate). Inspecting the images closely reveals that the melting events start at the veins. For instance, at −10°C a lot of liquid droplets are seen along the veins, whereas there are much less little droplets in the other regions of the the frozen drop. At −4°C the molten droplets are much more homogeneously distributed over the whole sample. That is, as expected from thermodynamics the melting event of ice crystals starts in the locations of highest solute concentrations, specifically in the veins containing the FCS. With the addition of 125 mM sucrose the first melting event shifts by about 3°C to lower temperature, consistently seen in the OCM images (Figs. 10 and 11) and the DSC measurements (Fig. 9).

## Discussion

MFI data of the samples frozen at −80°C and thawn to room temperature show a correlation between position in the freezing box and the extent of particle formation. In the middle of the freezing box vials are freezing and thawing slower than vials placed at the edge due to the temperature gradient driven by the passive heat transfer. This experimental set-up proves that cooling/heating rates have an impact on the number of nuclei, the ice crystal size and the amount and size of particles (Figs. 3 and 4). Slow cooling rates lead to fewer but bigger (ice) crystals (53), and this in turn results in fewer but bigger (protein) particles (Figs. 3 and 4). Thus, in terms of prevention of aggregate formation the best position for samples is in the middle of the freezing box. This is notwithstanding the fact that the number of particles not only depends on position in the freezing box, but also on the protein formulation itself. Each FT-cycle that the protein formulation is undergoing is leading to an increase of particle number due to repeatedly exposing the protein to freezing/thawing stress. This phenomenon can also be observed for the experiment series in which the protein concentration is varied. With increased protein concentration the number of small particles detected increases but the amount of bigger particles decreases.

The probability of particles growing into larger ones is smaller in sucrose solutions (54). Sucrose as a cryoprotectant seems to have an ambivalent effect on the protein solution in terms of particle formation. This additive is contraproductive, i.e., induces particle formation, in formulations containing low protein concentration. However, it has the positive effect of hindering the formation of particles for high protein concentration (30 mg/ml and 40 mg/ml). The desired cryoprotectant effect just occurs at higher (above 10 mg/ml) protein concentrations wherein the sucrose protects the protein from the ice surface. A possible reason for particle formation in 1 mg/ml formulations could be the excess of sucrose compared to protein concentration and hence simply the wrong sugar/protein ratio. That is, our data suggest there is an ideal ratio of sucrose to protein on a U-shaped curve describing cryoprotection ability.

DLS measurements indicate a monodisperse particle distribution in the protein formulation without sucrose regardless of how high the protein concentration is (Table I). Also after the addition of sucrose monodispersity remains as judged from the low PdI. The only exception is for the 1 mg/ml protein solution, for which the addition of sucrose increases the PdI by a factor of 10–40. This indicates heterodispersity and agglomeration, presumably of the sucrose itself. At the same time the hydrodynamic diameter remains unaffected at 11.2 nm, which corresponds to the mononomeric mAb. In other words, the sucrose does not interact with the mAb at 1 mg/ml. By contrast, for solutions containing 10 mg/ml or more of mAb an increase of 2r by 1 nm ± 0.3 nm is noticed after addition of sucrose. We rationalize this finding by a competition of water and sucrose molecules for their presence at the protein surface (55). Following the preferential exclusion mechanism (56–58) the larger sugar molecules are excluded from the protein surface, even though this is thermodynamically not favorable. This results in decreased protein solubility (55) and protein molecules being increasingly hydrated (59). In order to compensate for the unfavorable thermodynamics the surface area of the protein has to decrease, which is achieved by agglomeration and a possible reason for the increase of 2r in Table I. In other words, the presence of sucrose may destabilize the protein’s colloidal state (55).

The increase of 2r upon increasing protein concentration (Table I) is explained by the limited ability of particle diffusion in the citrate buffer, which decreases with an increase in concentration (»negative dynamic interaction parameter«). That means that during freezing particles move slower (slower diffusion) and closer together which finally leads to an increase of hydrodynamic diameter of the aggregates due to the binding effect of the mAb with the cosolutes present (55).

The OCM images show that the pure buffer, frozen at slower rates of 1–2°C/min down to −80°C, forms multiple ice crystals that originate from different parts of the droplet. The see-through areas can be traced back to pure frozen water. Due to the insolubility of salts in ice (60), excipients and other co-solutes get excluded and remain in the liquid phase (22). The ice crystals form a freeze-front that slowly, but progressively pushes the undisolved components as well as the protein into the direction of crystal growth (37,61). Channels with higher sodium citrate concentration are formed which are visible in the images (Fig. 6). Such concentration changes may lead to pH changes and destabilization of the protein. (62) For faster cooling rates the ice does not have enough time to push the solute away, so that entrapment of the undissolvable components takes place, which ultimately results in a more homogeneous distribution. Instead, slower rates favor the freezing process to start from multiple nucleation sites which leads to ice crystal growing in a platelet–like structure orientated in various angles (Fig. 6). The impact of sucrose is to reduce the number of nucleation events, and thus the number of ice crystals, and at the same time the high concentration of sucrose serves the purpose of keeping protein molecules apart, thereby preventing aggregation. An important finding is that sucrose addition and slow cooling rates favor an exothermic freezing event of the FCS over the vitrification of the FCS. That is, only with sucrose and at slow cooling rates, also crystals form inside the veins and interdentritic spaces, resulting in a more homogeneous appearance in OCM images. By contrast, without sucrose or at faster rates the FCS turns into glass, so that glassy parts and crystalline ice co-exist. It is not entirely clear from our data whether the second freezing event should be avoided or not – it would be desirable to investigate the number and size of particles for the cases of crystallization and vitrification of the FCS.

DSC results show that freezing temperatures of the sample (buffer with and without sucrose) vary by up to 10°C, as expected from the stochastic nature of the freezing process and classical nucleation theory (63). The determination of T_g_’ of the freeze-concentrated sodium citrate solution had been difficult because of the low concentration (25 mM) and limited sensitivity of the DSC instrument. By employing faster scan rates the signal of the DSC is intensified, which enabled us to determine T_g_’ to be −40°C at 40°C/min. The addition of 125 mM sucrose allows for detection even at slow heating rates, and shifts T_g_’ to about −32°C. This indicates about 70 wt% of sucrose in the vitrified freeze-concentrated solution. The vitrification of the freeze-concentrated pure sodium citrate buffer is in agreement with literature data (64). Some of the heating curves of the pure buffer show a weak exotherm before the main melting peak of the solution (circled area in Fig. 9). This indicates solute crystallization, also called cold-crystallization. Such cold-crystallization events should be avoided to ensure product quality – which is the case after addition of sucrose, even for slow thawing experiments. Possibly, this is so because sucrose adsorbs on the ice surface through hydrogen bonds between the hydroxyl groups and the ice lattice (65), therebye preventing its crystallization. This coating of the edges of ice might be at the origin of the smoothing of the edges seen in Fig. 7 for slow heating experiments. Without sucrose this can only be avoided by thawing at rates of 20°C/min or higher. OCM images indicate that freeze-concentrated solution melts long before ice thaws, which enables cold-crystallization in between the ice crystals. This is detected by the DSC and shown as a kink in the thermograms (Fig. 9).

T_g_’ of sucrose solutions is observed at around −35°C (66,67) and shifts to higher temperatures when the heating rate is increased (see Table II), in accordance with literature data (68). Our data is in agreement with the data provided by Levine and Slade, who determined T_g_’ at −35°C (69). We emphasize that we do not see a second glass transition in our DSC scans near −42°C, as was the case in earlier literature (70–72). However, Fig. 8 shows that a second crystallization process takes place upon cooling the sample beyond T_f_ for sucrose-containing samples at slow cooling rates. This second freezing event can be traced back to the solidification of freeze-concentrated solution which has been trapped in between the ice crystals after the first freezing event.

## Conclusion

The freezing and thawing of pharmaceutical protein solution has proven to be a very complex and delicate process. We have measured particle number of protein samples after changing parameters like composition of the buffer formulation, sample position in the freezing box, number of FT-cycles and protein concentration by using MFI, Archimedes and DLS. Additionally, the buffer was analysed with and without sucrose at different freezing and thawing rates by OCM and DSC. Generally, in terms of particle number and hence aggregate formation it is best to avoid multiple FT-cycles. Slow freezing and slow thawing rates favor the formation of bigger but fewer aggregates whereas fast freezing and fast thawing rates lead to more, albeit smaller aggregates. The cryoprotective-effect of sucrose is confirmed at higher protein concentrations (above 10 mg/ml) and even then it mostly prevents particle formation of micron and visible particles. A reduced number of smaller particles (above 0.2 μm) could be seen only after one FT-cycle, after 5 FT-cycles no significant difference of particle number between formulation with and without sucrose was detected. Besides, sucrose has been proven to be contra-productive at 1 mg/ml protein concentrations which is supported by data retrieved by DLS. The optimal protein: stabilizer (sucrose) ratio has to be found in order to achieve the desired stabilizing effect, since sucrose can lead to conformational stabilization on one side but also destabilization on the other side. Therefore, the optimal formulation should improve both, solubility and kinetic stability of the protein (55). Particle size distribution data confirm particle concentration reduction in samples with 30 mg/ml protein concentration. In order to determine the optimal protein concentration regarding aggregate number reduction higher protein concentrations have to be tested (18). Further investigation is necessary to determine the impact of highly concentrated mAb formulations.

OCM data support the statement that fast freezing rates (above 30°C/min) induce microscopic scale freeze-concentration, i.e., the solutes get trapped in between the formed ice crystals. Also, slow freezing rates of 1–2°C/min lead to a more uniform distribution of solutes in the frozen buffer droplet and hence limits concentration of solutes on the microscopic scale. The addition of sucrose shifts the melting and freezing temperatures down by 3°C. More importantly, the addition triggers the crystallization (rather than vitrification) of the freeze-concentrated solution (FCS) for slow cooling rates. This secondary freezing leads to a more homogeneous appearance of the frozen solution. Upon heating, cold-crystallization effects were seen to be suppressed in the presence of sucrose. The extra exotherm around −10°C observed for buffer solutions without sucrose until 0°C disappears when sucrose is added. Our OCM and DSC data do not yet permit making reliable extrapolations of cryo-concentration to the macroscopic scale because experiments of larger volumes are requiered.

In conclusion, aggregation as a freezing-damage is avoided best by applying slow freezing rates around 1–2°C/min and avoiding multiple FT-cycles. Additionally, sucrose suppresses cold-crystallization and particle formation especially when using fast freezing rates. Higher protein concentrations have proven to be advantageous in terms of particle formation prevention. It has to be said that our results apply only to our specific mAb formulation, i.e., other mAb formulations can behave differently and, therefore, require other handling.

## Abbreviations

DLS: Dynamic light scattering
DSC: Differential scanning calorimetry
FT: Freeze and thaw
mAb: Monoclonal antibody
MFI: Micro flow imaging
OCM: Optical cryomicroscopy
PdI: Polydispersity index
SEC: Size exclusion chromatography
T_f_: Freezing temperature
T_g_: Glass transition temperature
